# Interaction of integrin α_v_β_3_ and fibronectin under fluid shear forces: implications for tumor cell adhesion and migration

**DOI:** 10.3389/fcell.2025.1512672

**Published:** 2025-02-13

**Authors:** Paimin Zhuo, Quhuan Li, Bishan Yang, Na Li, Zhiqing Luo, Fengxia Zhang

**Affiliations:** ^1^ School of Bioscience and Bioengineering, South China University of Technology, Guangzhou, China; ^2^ Institute for Stroke and Dementia Research, Ludwig-Maximilians-Universität München, Munich, Germany; ^3^ Department of Nephrology, First Affiliated Hospital of Gannan Medical University, Ganzhou, Jiangxi, China

**Keywords:** integrin α_v_β_3_, fibronectin, tumor cell adhesion, force, catch bond

## Abstract

The interaction between integrin α_v_β_3_ and fibronectin enables tumor cell adherence to endothelial layers under diverse hydrodynamic blood flow conditions, particularly in low shear stress regions. Understanding the mechanical binding characteristics between integrin α_v_β_3_ and fibronectin under different hydrodynamic environments can provide insights into tumor cell invasion and proliferation. Here, the adhesive behavior of fibronectin-functionalized microspheres on integrin α_v_β_3_-coated substrates under various wall fluid shear forces (0.1–0.7 dyn/cm^2^) was assessed using a parallel plate flow chamber system. The bond lifetimes of integrin α_v_β_3_-fibronectin initially increased and then decreased, indicating transition from a “catch bond” to “slip bond.” Upon perfusion of fibronectin-coated microspheres into flow chambers with high-density integrin α_v_β_3_ coating, the rolling velocity of the microspheres increased with increasing shear force. Additionally, the mean stop time and stop frequency exhibited a force-dependent biphasic pattern, initially increasing and then decreasing with shear force, demonstrating a nuanced response to mechanical forces. Thus, the integrin α_v_β_3_–fibronectin interaction displays a “catch bond” property, influencing cell distribution in varying fluid shear forces by promoting optimal adhesion in specific shear sites. These insights enhance our understanding of tumor cell adhesion and migration in hydrodynamic environments and may aid the design of integrin α_v_β_3_-targeted therapies.

## 1 Introduction

In dynamic hydrodynamic environments, the adhesion and migration of tumor cells along vessel walls are pivotal events in tumor invasion (Chen et al., 2022; Fares et al., 2020). The specific expression of integrins on the surface of tumor cells significantly alters the adhesive behavior of the cells within the fluid environment, facilitating their invasion from apoptosis (Haeger et al., 2020; Janiszewska et al., 2020; Lamb et al., 2018; Xiong et al., 2021). Integrins play crucial roles in cellular responses to extracellular stimuli and in the physical attributes of blood flow, thereby influencing cellular interactions vital for tumor development and progression (Li et al., 2021; Wong et al., 2020). Furthermore, interactions between integrins and their ligands [e.g., fibronectin (FN)] enable mechanochemical signal transition, which is crucial for many cellular processes such as proliferation, migration, and cell division (Bonin et al., 2022; Wu et al., 2023). Integrin-based adhesion malfunctions are involved in various pathological conditions, including cancer (Cooper and Giancotti, 2019), fibrosis (Meagher et al., 2021), and inflammatory diseases (Slack et al., 2022). Several integrin-targeting inhibitors, including small molecules and antibodies that mainly target inflammatory diseases, have passed clinical trials and are now commercially available (Slack et al., 2022), indicating that integrin targeting might be a promising therapeutic approach in the near future. Despite considerable efforts to develop integrin inhibitors for cancer therapy, most of these inhibitors have failed because of their lack of efficacy (Szpak et al., 2023; Pachane and Selistre-de-Araujo, 2024). Therefore, investigating the mechanism of the binding behavior in different fluidic environments could provide further insights for designing the structure of integrin inhibitors and pharmaceutical carriers.

At the model level, the interactions governing integrin-mediated adhesion display intricate catch (increased bond lifetime with force) and slip (decreased bond lifetime with force) behaviors in response to varying forces (Yago et al., 2004; Yago et al., 2007; Zhu et al., 2008). Initially, catch-slip bond behavior was experimentally demonstrated in the interaction between P-selectin and PSGL-1 (Marshall et al., 2003). Subsequently, in the 2010s, more catch bonds were discovered, mainly in adhesive molecules, including integrins. One example of such catch bond behavior included the binding between integrin alpha IIb beta 3 and FN or fibrinogen (Chen et al., 2019). Notably, among integrins, the conformational changes of integrin α_v_β_3_ was demonstrated to be force-dependent (Chen et al., 2017). Additionally, a computational study showed that the force-dependent interaction between integrin α_v_β_3_ and FN trigger the activation of integrin α_v_β_3_ by changing its conformation (Wang et al., 2017). Despite knowing the structure of integrin α_v_β_3_ for decades, the mechanism underlying its binding with FN under diverse fluidic conditions remains elusive.

Integrin α_v_β_3_ is a transmembrane protein comprising three main regions: cytoplasmic domain, transmembrane domain, and extracellular domain (Van Agthoven et al., 2014; Xiong et al., 2002). The cytoplasmic domain, which is connected to the cytoskeleton, is an important region for transmitting cell signals (Xiong et al., 2002). The extracellular domain of integrin α_v_β_3_ is the active domain engages its ligand, leading to activation of the integrin and transmission of the signal from the outside to the inside (Luo et al., 2007). The β-propeller domain of the α_v_ subunit and the β_I_ domain of the β_3_ subunit, located in the extracellular domain, constitute the binding site of its ligand (Xiong et al., 2009; Xiong et al., 2002). Integrin α_v_β_3_ recognizes several extracellular matrix proteins, including FN, vitronectin (Hsu et al., 2007), laminin subtypes α1, α2, and α5 (Genersch et al., 2003), and collagen types I-III (Zeltz et al., 2014). Among these ligands, FN is considered a key protein that mediates tumor cell invasion. FN interacts with integrin α_v_β_3_ through its peptide motif Arg-Gly-Asp (RGD) domain, leading to tumor cell proliferation, adhesion, migration, and invasion (Dolinschek et al., 2021; Takada et al., 2021). In the study of Elosegui-Artola et al., atomic force microscopy and micropipette technology were used to measure the lifetime of a single fibronectin bond with α_v_β_3_, and it was found that there was a catch bond between fibronectin and integrin α_v_β_3_ (Elosegui-Artola et al., 2016). Notably, integrin α_v_β_3_ also plays a role in angiogenesis (Makowski et al., 2021) and has been identified as a biomarker in various tumor types (Echavidre et al., 2022; Shi et al., 2019) and angiogenesis-related diseases, underscoring its significance in disease biology and therapeutics. The interaction between integrin α_v_β_3_ and its primary ligand RGD, inherent in FN, governs several tumor cell-related biological processes, including cell migration, cell invasion, angiogenesis, and cell proliferation (Gu et al., 2023). Since the interaction between integrin α_v_β_3_ and RGD regulates various cell biological functions and integrin α_v_β_3_ is specifically expressed on some kinds of tumor cells, some integrin α_v_β_3_-targeted RGD analogs have been designed to detect and treat tumor cells (Burke et al., 2002; Gajbhiye et al., 2019; Lode et al., 1999; MacDonald et al., 2001; Sharma et al., 2021; Turaga et al., 2021). For example, cilengitide was designed to target integrin α_v_β_3_ to treat cancer, but was eventually not released in the market due to its low efficacy (Burke et al., 2002). In this case, investigating the interaction between integrin α_v_β_3_ and RGD is essential and can provide references for α_v_β_3_-targeted drug design.

This study delves into the detailed adhesive behaviors of FN-coated microbeads interacting with integrin α_v_β_3_-functionalized plates, studying the tethering and rolling of these beads under distinct shear stresses using the parallel-plate flow chamber (PPFC). Understanding the intricacies of this interaction under different fluidic conditions can inform the design of integrin-targeted inhibitors and pharmaceutical carriers, thereby offering a promising avenue for future therapeutic interventions.

## 2 Materials and methods

### 2.1 Proteins

The proteins analyzed in this study are described below. The integrin heterodimer α_v_β_3_, obtained from R&D Systems (Minneapolis, MN, United States; Catalog #3050-AV-050), consists of an α_v_ extracellular subunit (110.5 kDa) and a β_3_ extracellular subunit (80.8 kDa) linked by covalent bonding of disulfide bonds. The N-terminal head region is the ligand binding site of FN, formed by combination of the β-propeller structure of α_v_ subunit and vWF-A domain of β_3_ subunit. The FN protein (R&D Systems, Minneapolis, MN, United States; Catalog #1918-FN-02M) was derived from human plasma. The FN used in this study was made up of three types of homologous structural repeats, termed FN type I, type II, and type III repeats (EDA and EDB). A truncated fibronectin 1.3 (FN1.3; R&D Systems, Minneapolis, MN, United States; Catalog #3938-FN-050), which includes the EDB plus type III domains #8–13 and the initial region of domain 14, was also used; these domains facilitate association with α_v_β_3_. Furthermore, the RGD motif, located between type III domains #9 and #10, determines the affinity of FN to integrin α_v_β_3_.

### 2.2 Coupling of integrin α_v_β_3_ with flow chambers and FN with microspheres

To couple integrin α_v_β_3_ to flow chambers, a square region (25 mm^2^) coated with proteins was labeled in the center of each 35 mm dish well (Corning, United States) and was constrained with a clean silicon rubber. Then, 40 μL of integrin α_v_β_3_ was added onto the square region in the center of the Petri dish, and directly adsorbed onto the bottom via incubating it at 4°C refrigerator for 16 h (Li et al., 2012). Here, different concentrations of integrin α_v_β_3_ were chosen for specific experimental purposes. Based on the results of a preliminary experiment with various concentration gradients, 20 ng/mL and 40 ng/mL of integrin α_v_β_3_ were used to investigate transient tethers and rolling adhesion, respectively. After overnight incubation, the coated square region was washed three times with phosphate-buffered saline (PBS) to remove the unadhered proteins, and then the 35 mm dish bottom was incubated with 800 μL PBS containing 2% bovine serum albumin (BSA) at room temperature for 30 min to prevent non-specific adhesion.

To coat microspheres with FN, glass beads of 3 μm radius (BD Warrington, PA) were washed three times with 1 mL PBS, followed by functionalization of the microspheres by mixing the beads with 40 μL of FN (200 μg/mL) or FN1.3 (100 μg/mL). To mix the microspheres and proteins well, they were incubated in a falcon rotating every 10 min for 2 h in a 4°C refrigerator overnight. Afterwards, the beads with protein were washed once and blocked for 2 h with PBS containing 2% BSA. Thereafter, the beads were stored at 4°C in PBS containing 0.1% sodium azide for up to 5 days (Yago et al., 2007).

### 2.3 Flow assays

FN- or FN1.3-expressing microspheres (0.5 × 10^6^ beads/mL in HBSS, containing 1% BSA, with calcium and magnesium ions at a concentration of 1 mM) were perfused under various flow shear stress τ_w_ (0.1–0.7 dyn/cm^2^) over circular flow chamber (GlycoTech, Gaithersburg, Maryland) with low or high density of integrin α_v_β_3_. A silicon rubber gasket was used to create a working flow space with length × width × thickness = 2 × 0.5 × 0.0254 cm^3^ (Li et al., 2016; Yago et al., 2004). The flow shear stresses were converted into flow shear rates and controlled using a Harvard pump. The cell adhesion process was observed using a Zeiss inverted microscope and adhesion images were recorded using a CMOS camera at a speed of 100 frames per second (fps). After transferring the imaging data into video, the rolling trajectory of the cells was tracked using IPP software (Image Pro Plus). To change the viscosity in specific experiments, 3% (w/v) ficoll (3–5 × 10^5^ Mr; Amersham Biosciences) was added to the medium to increase the viscosity by 1.69-fold, as determined using a cone plate viscometer (Yago et al., 2007). To certify all tethering and rolling events were mediated by specific interaction of integrin α_v_β_3_ with FN, HBSS with 2% BSA were added to the incubated substrate to exclude non-specific adhesion of microspheres.

### 2.4 Tether lifetime measurement

Transient tether lifetimes of one stop 3 μm-radius beads bearing FN or FN1.3 were measured on low densities of integrin α_v_β_3_ (coated at 20 μg/mL) that did not support rolling or skipping at any wall shear stresses (Li et al., 2012; Yago et al., 2004). Images of the transient tethers were captured using a high-speed complementary metal-oxide speed CMOS acquisition system (Mikrotron GmbH MC 1310; Norpix Ins.) at 100 fps (Li et al., 2012). The replayed images were captured at a slow speed, and the duration of the transient tethers were recorded through frame-by-frame analysis with IPP. Three sets of lifetimes (approximately 60 tethering events per set) were measured for each wall shear stress. Data are presented as mean ± standard deviation of average lifetimes. When the tether lifetimes were produced by one bond interaction of integrin α_v_β_3_ with FN/FN1.3, first-order dissociation kinetics model can be applied to perform linear fitting for off rates (Coburn et al., 2011; Li et al., 2016). Therefore, off-rates can be derived from negative slope by analyzing the plot of ln (number of events with a lifetime ≥ *t*) vs. *t*, which was fitted using a straight line. The correlation coefficient *R* ^2^ was >0.9 for all fits. The means and standard deviations of the off rates at each wall shear stress were calculated (Li et al., 2012; Sarangapani et al., 2004).

### 2.5 Rolling behavior analysis

To determine the rolling behavior, we first observed the cells rolling with a ×10 objective on a Zeiss microscope and captured images with a high-speed camera at 100 fps. The Image Pro Plus software (IPP, Media Cybernetics, v6.0) was used to track the rolling cells and obtain the coordinates of the centroids. The instant velocity was calculated by dividing the distance between two continuous centroids by their elapsed times (10 ms). The continuous sliding average of every 10 frames for raw instant velocity data was obtained to reduce the noise levels. Custom-designed Excel macros were used to analyze the rolling step for the beads. Microsoft Excel macros were written based on a minimal model that describes a continuous stop-and-go motion mediated by only one and two bonds (Yago et al., 2004). As soon as a bond dissociated in the rear region of the bead, the bead entered the acceleration phase. If a new bond was formed in the front region of the bead, the bead entered the deceleration phase. Here, a step was defined as a cycle of acceleration and deceleration, which may or may not include a stop phase. Based on this model, several researches have conducted rolling step analysis for rolling cells or microspheres mediated by L-selectin-PSGL-1 interaction (Yago et al., 2004), E-selectin-ligand interaction (Li et al., 2012; Li et al., 2016), and vWF-GPIbα interaction (Coburn et al., 2011). In this study, we analyzed the rolling behavior of FN/FN1.3-coated bead on integrin α_v_β_3_ substrate. A stop phase was defined for rolling bead when the bead’s instantaneous velocity was lower than the system noise level (<20 μm/s) measured from settled beads in a control preliminary experiment. An acceleration and deceleration threshold of ±600 *μ*m/s^2^ was selected based on specific formation and breakage of bonds measured for free flowing cells caused by Brownian motion. More than 800 stop-and-go events were recorded from approximately 45 beads for each shear stress. Finally, several parameters were extracted from these stop-go events, including the mean stop times and stop frequencies.

### 2.6 Statistical methods

Statistical significance was evaluated using GraphPad Prism (version 9.5.0) and Origin (2017). All data are expressed as mean ± standard deviation (SD) in the figures. Each group underwent three independent replicate experiments, with a minimum of 20 microspheres collected per experiment. Statistical analyses were performed using Student’s t-test and one-way ANOVA with multiple comparisons to assess the significance between groups (ns for *p* > 0.05, * for *p* < 0.05, ** for *p* < 0.01, *** for *p* < 0.001, **** for *p* < 0.0001). Prior to applying these parametric tests, we conducted the Shapiro-Wilk test to verify the normality of the data and Levene’s test to check for homogeneity of variance.

## 3 Results

### 3.1 Specific experiment

**FIGURE 1:**
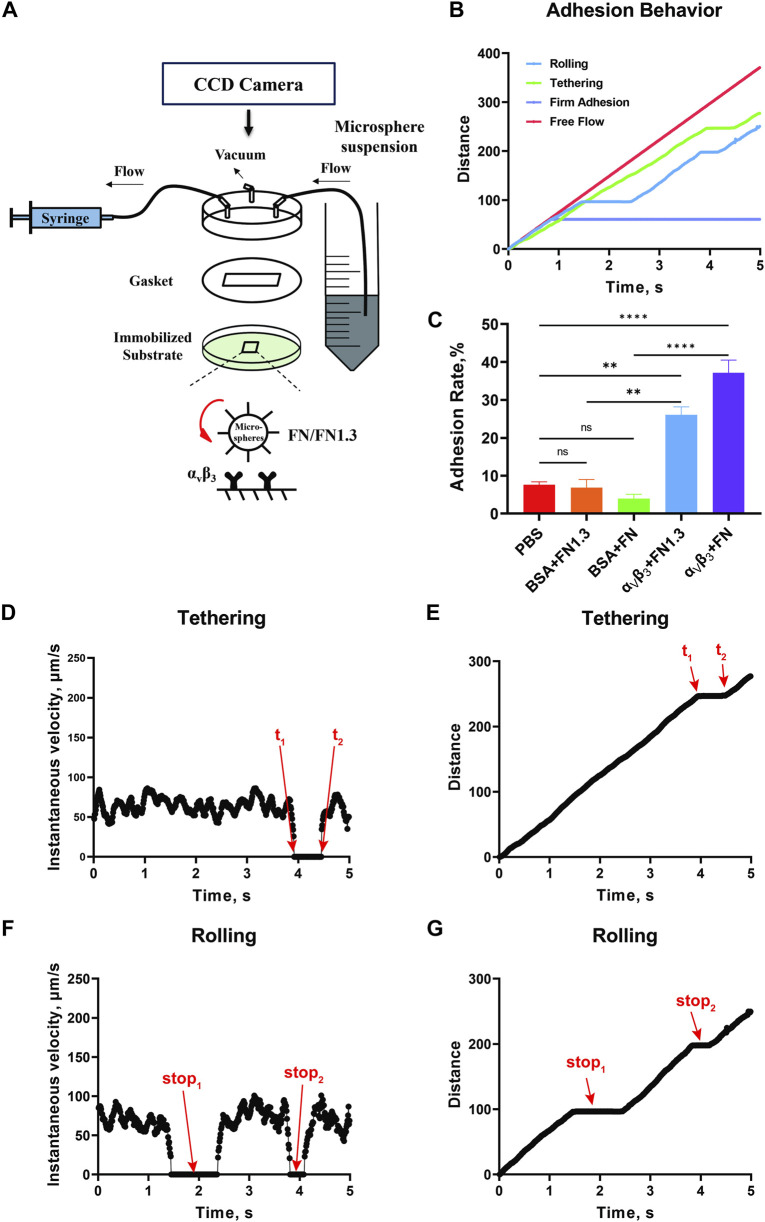
Schematic of the parallel plate flow chamber and specific experiment. **(A)** The flow chamber was assembled by a lexan, a gasket (length × width × thick = 2 × 0.5 × 0.0254 cm^3^) and 35 mm dish. There were three channels on the Lexan, including inlet, outlet, and vacuum. **(B)** The distance-time curve tracked by Image Plus Pro (IPP) software. Lifetime was calculated according to the upper graph from t_1_ to t_2_ **(C)** Adhesion rate of fibronectin (FN)/truncated fibronectin 1.3 (FN1.3) functionalized microspheres on phosphate-buffered saline (PBS) adn bovine serum albumin (BSA)-blocked, and integrin α_v_β_3_-immobiled substrates. Data represent the mean ± SD of three experiments. The significance of the difference is shown by *p*-value, with ns. for *p* > 0.05, and ** for *p* < 0.005. **(D, E)** Instantaneous velocity and cumulative distance of one tethering event. **(F, G)** Instantaneous velocity and cumulative distance of one rolling event.

After setting up the flow chamber as shown in Figure 1A, binding experiments were performed to test the adhesion specificity of the integrin α_v_β_3_-FN interaction. We perfused the FN-expressing beads into the flow chamber to give the beads the opportunity to adhere to the integrin α_v_β_3_ substrate at flow shear stress 0.3 dyn/cm^2^. The rolling, tethering, and firm adhesion events were determined based on distance-time curves (Figures 1B, C). Tethering refers to the transient stop event mediated by a single molecular interaction (Figures 1D, E). Rolling refers to the occurrence of more than two consecutive molecular interactions following the “stop-go-stop” pattern (Figures 1F, G) (Kong et al., 2009), and the firm adhesion refers to the stable adhesion occurring after the interaction of multiple pairs of molecules, with durations exceeding 3 min (Figure 1B). The percentage adhesion rate was calculated by dividing the number of the three types of adhered cells (Figure 1C) by the number of all flowing cells in the same window and time. The results showed that the adhesion rate was very low with approximately 5%–10% of FN or FN1.3-captured beads adhering to PBS or 2% BSA-coated substrates. However, the percentage of adhesion rate was increased to approximately 30% and 40% for FN1.3- and FN-coated beads adhered on integrin α_v_β_3_ substrates, respectively. Therefore, the tethering and rolling and firm adhesion events in our experiment system were specific for FN- or FN1.3-coated beads because they were eliminated by blocking with PBS containing 2% BSA (Figure 1C).

### 3.2 Interaction of integrin α_v_β_3_ with FN forms a catch-slip bond with shear stress force

**FIGURE 2:**
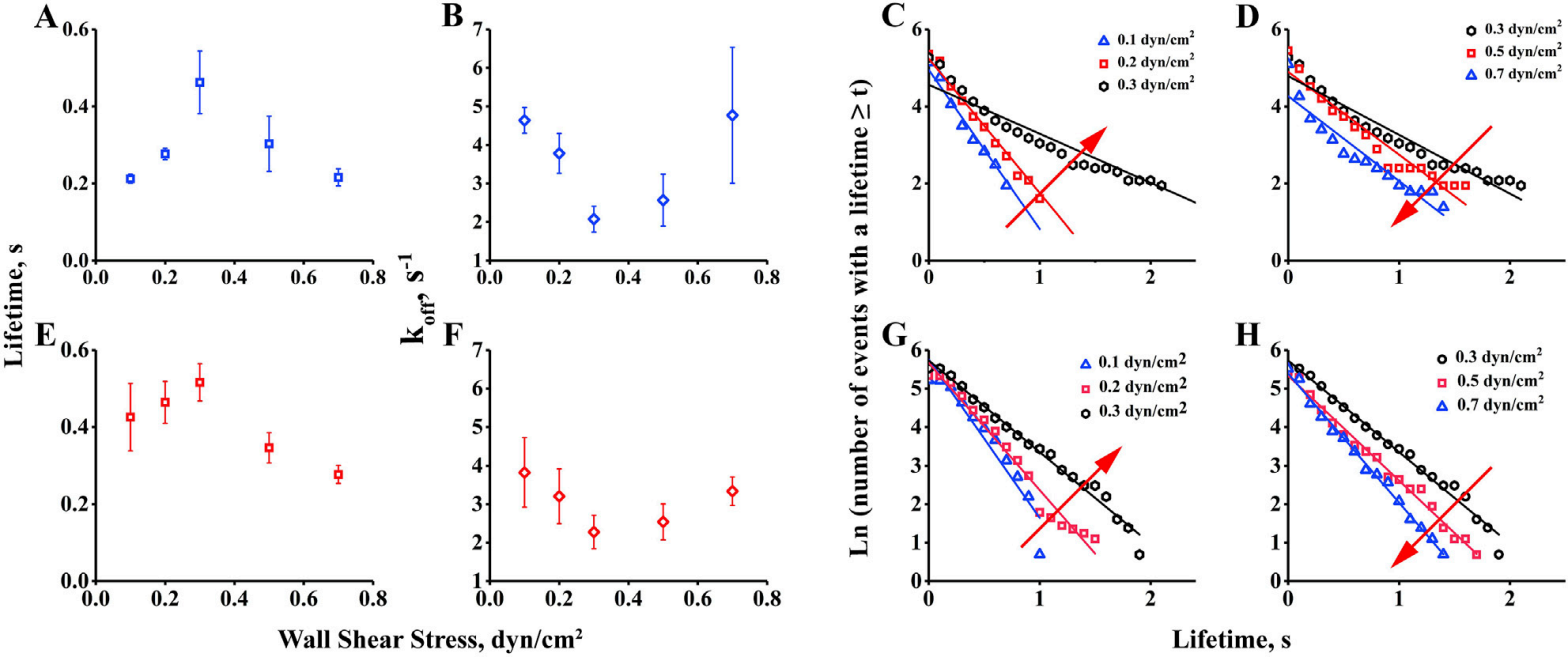
Measurement of transient tether lifetime and dissociation rate constant, *k*_*off*_. Lifetimes of transient tethers of fibronectin (FN) and truncated fibronectin (FN1.3)-bearing beads **(A, E)** on a surface with low-density integrin α_v_β_3_ were plotted against wall shear stress. The dissociation rate constant, *k*_*off*_ **(B, F)**, derived from negative slope by linear fitting the tether lifetime plot of ln (number of events with a lifetime ≥ t) vs. t for low shear stress 0.1–0.3 dyn/cm^2^ **(C, G)** and high shear stress 0.3–0.7 dyn/cm^2^ **(D, H)**. Data represent the mean ± SD of three experiments.

To investigate the influence of shear force on the interaction of integrin α_v_β_3_ with FN, we measured transient tether lifetimes on bottom of the chamber with low-density of integrin α_v_β_3_ (coated at 20 μg/mL). These densities did not allow the FN-bearing beads to roll or skip on the substrate. Only a few sliding cells had the opportunity to transiently and briefly stop on the substrate. We used a high-speed camera to record these events at 100 fps, and the transient tether lifetimes were obtained by analyzing the duration of the stop events by offline tracking. The results indicated that the transient tether lifetime first shortened, then prolonged with increasing wall shear stress, exhibiting catch-slip bonds in the interaction of integrin α_v_β_3_ with FN (Figure 2A) and FN1.3 (Figure 2E). This biphasic lifetime curve has previously been reported for selectin family member E/P/L-selectin interacting with PSGL-1 (Li et al., 2012; Marshall et al., 2003; Yago et al., 2004), as well as integrin family member α_L_β_2_ interacting with ICAM-1 (Chen et al., 2010), and immunoglobulin superfamily member TCR interacting with pMHC (Depoil and Dustin, 2014). Furthermore, we used the transient tether lifetimes to derive the dissociation rate constant off-rates (*k*_*off*_), which can be reported as the dissociation ability of single bonds. By linear fitting curve of ln (number of events with a lifetime ≥ *t*) vs. *t* for low shear stress 0.1–0.3 dyn/cm^2^ (Figures 2C, G) and high shear stress 0.3–0.7 dyn/cm^2^ (Figures 2D, H), the off-rates were extracted from the negative slope of the fitting plots. As shown in the figure, the off-rates decreased initially, then increased finally with wall shear stress in the tethers mediated by both integrin α_v_β_3_-FN (Figure 2B) and integrin α_v_β_3_-FN1.3 (Figure 2F). The biophasic transient tether lifetime curve was inversely related to the dissociation rate constant off-rate, indicating that the force-enhanced bond lifetime was due to force-reduced bond dissociation. The lifetime and off-rate curves had the same tendency for both FN and FN1.3, indicating that FN1.3 containing #8–14 domains was sufficient for the interaction with integrin α_v_β_3_.

### 3.3 Biphasic rolling-velocity pattern of beads mediated by interaction of integrin α_v_β_3_ with FN

**FIGURE 3:**
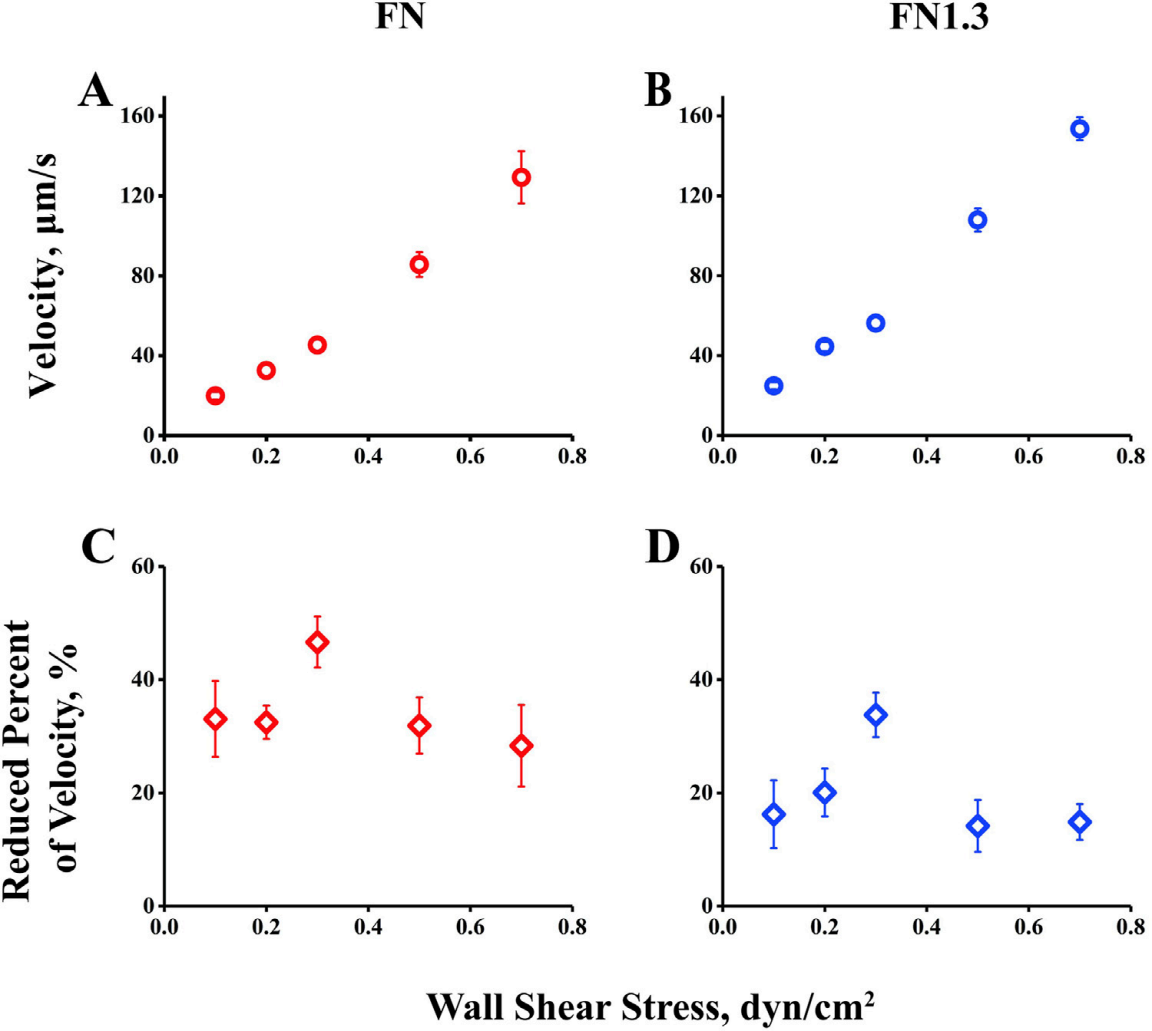
Rolling velocity and reduced percent of rolling velocity of beads by interaction of integrin α_v_β_3_ with fibronectin (FN) or truncated fibronectin (FN1.3). **(A, B)** Velocity of FN-coated beads (blue square) and FN1.3-coated beads (red square) rolling on integrin α_v_β_3_- immobilized bottom. **(C, D)** The reduced percent of rolling velocity of FN-coated beads (red diamond) and FN1.3-coated beads (blue diamond). The data were recorded at 100 frames per second, and the mean ± SD of three independent experiments was presented.

First, we measured mean rolling velocity of beads rolling on immobilized integrin α_v_β_3_ for 10 s and found it to change with increasing flow from the threshold to and above the optimal value. The mean velocity, a global parameter of rolling adhesion, increased monotonously with the wall shear stress from 0.1 to 0.7 dyn/cm^2^. At first glance, there was no catch bond phenomenon in this observation (Figures 3A, B). However, when we changed the vertical coordinate from rolling velocity to a reduced percentage of velocity, defined as the ratio of the decreased velocity (from free flowing to rolling) to the free-flowing velocity, the reduced percentage of the velocity first increased and then decreased as the wall shear stress increased (Figures 3C, D), similar to previous catch bond observations. This suggests that the interaction of integrin α_v_β_3_-FN increased the reduced percent of velocity, that is decreasing value of rolling velocity, to help stabilize rolling on the substrate in the catch bond region (0.1–0.3 dyn/cm^2^). Beyond the stable rolling optimum shear stress (0.3 dyn/cm^2^), the reduced percent of velocity decreased with shear stress in the slide bond region (0.3–0.7 dyn/cm^2^), where the beads rolled quickly.

### 3.4 Rolling velocity mediated by integrin α_v_β_3_-FN interaction under flow

**FIGURE 4:**
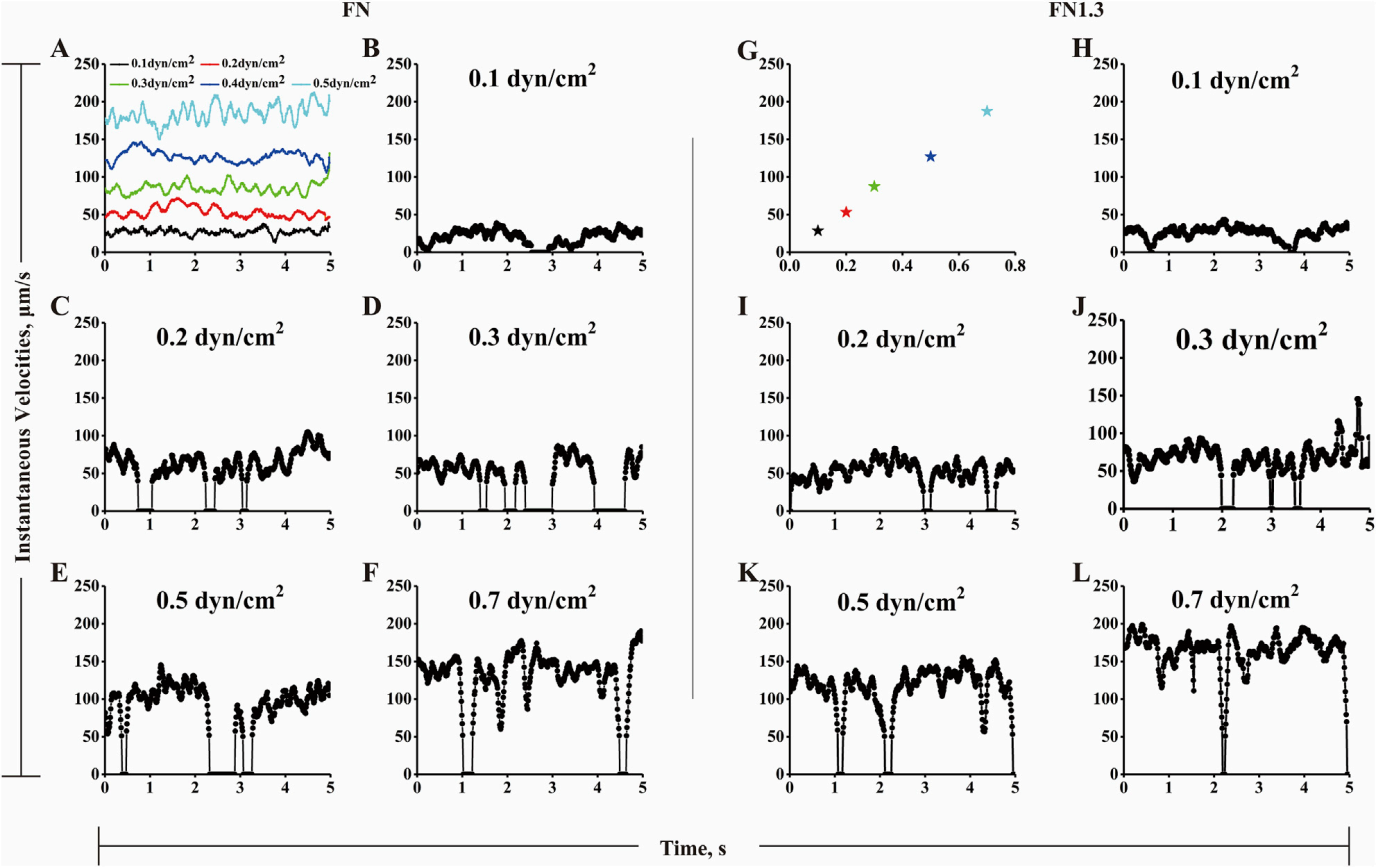
The instantaneous rolling velocity of beads by interaction of integrin α_v_β_3_-fibronectin (FN)/truncated fibronectin (FN1.3). **(A)** Shown are instantaneous velocities of five representative free flowing microspheres under various shear stress of 0.1 (black line), 0.2 (red line), 0.3 (green line), 0.5 (dark blue line) and 0.7 dyn/cm^2^ (wathet line). (B-F, H-L) Representative instantaneous velocities of FN **(B–F)**/FN1.3 **(H–L)** bearing microspheres of 3 μm radius flowing over and being arrested on a surface coated with integrin α_v_β_3_ (200 ng/mL) at wall shear stress below, equal and above the shear optimum (0.1, 0.2, 0.3, 0.5 and 0.7 dyn/cm^2^). The data were recorded at 100 fps. The instantaneous velocities were set as zero when their values were below 50 μm/s when the shear stress stronger than 0.2 dyn/cm^2^, while instantaneous velocities were set as zero when their values were 15 and 25 μm/s at 0.1 dyn/cm^2^ and 0.2 dyn/cm^2^, respectively. **(G)** Free flowing beads (pentagon) were referred to the sliding beads on the same focal plane, which has not any interaction with other molecular. The R value of the fitted curve is 0.9946.

The biphasic lifetime likely governs the biphasic mean rolling velocity by controlling the stop times between the two go phases. To test these hypotheses, we analyzed the rolling behaviors of FN-bearing microspheres on integrin α_v_β_3_-coated substrates recorded using high speed camera at 100 fps. The centroid coordinates of the microspheres were obtained by outline tracking frame-by-frame with IPP. Instantaneous velocity profiles were plotted by dividing the distance between two continuous centroid coordinates by the corresponding elapsed time. As shown in Figure 4, the irregular stop-and-go motions (Figures 4B–F) owning to specific FN-integrin α_v_β_3_ interaction were different from free-flowing microsphere motions (Figure 4A). As shear stress increased from 0.1 dyn/cm^2^ to 0.3 dyn/cm^2^, the flow force promoted the frequency of irregular motions with longer stop times. As shear stress increased beyond the optimal value of 0.3 dyn/cm^2^, the trend was reversed. With increasing shear stress from 0.3 dyn/cm^2^ to 0.7 dyn/cm^2^, the flow force reduced the frequency of irregular motions with shorter stop times. Similar rolling behaviors were observed in the FN1.3-bearing microspheres (Figures 4H–L). These rolling phenomena are possibly correlated with the lifetimes of integrin α_v_β_3_-FN bonds, and governed by catch bond mechanism. That is, a weak short-lived bond will become stronger and longer-lived as the flow force increases from a low level to an optimal value (a catch bond-dominating region) and then switch to a weak shorter-lived bond as the force increases across the optimum (a slip bond-dominating region). The transitions between catch and slip bonds govern the stop-and-go instantaneous velocities below and above the optimal force.

### 3.5 Shear stress force governs rolling mean stop times and stop frequencies below and above the flow optimum

**FIGURE 5:**
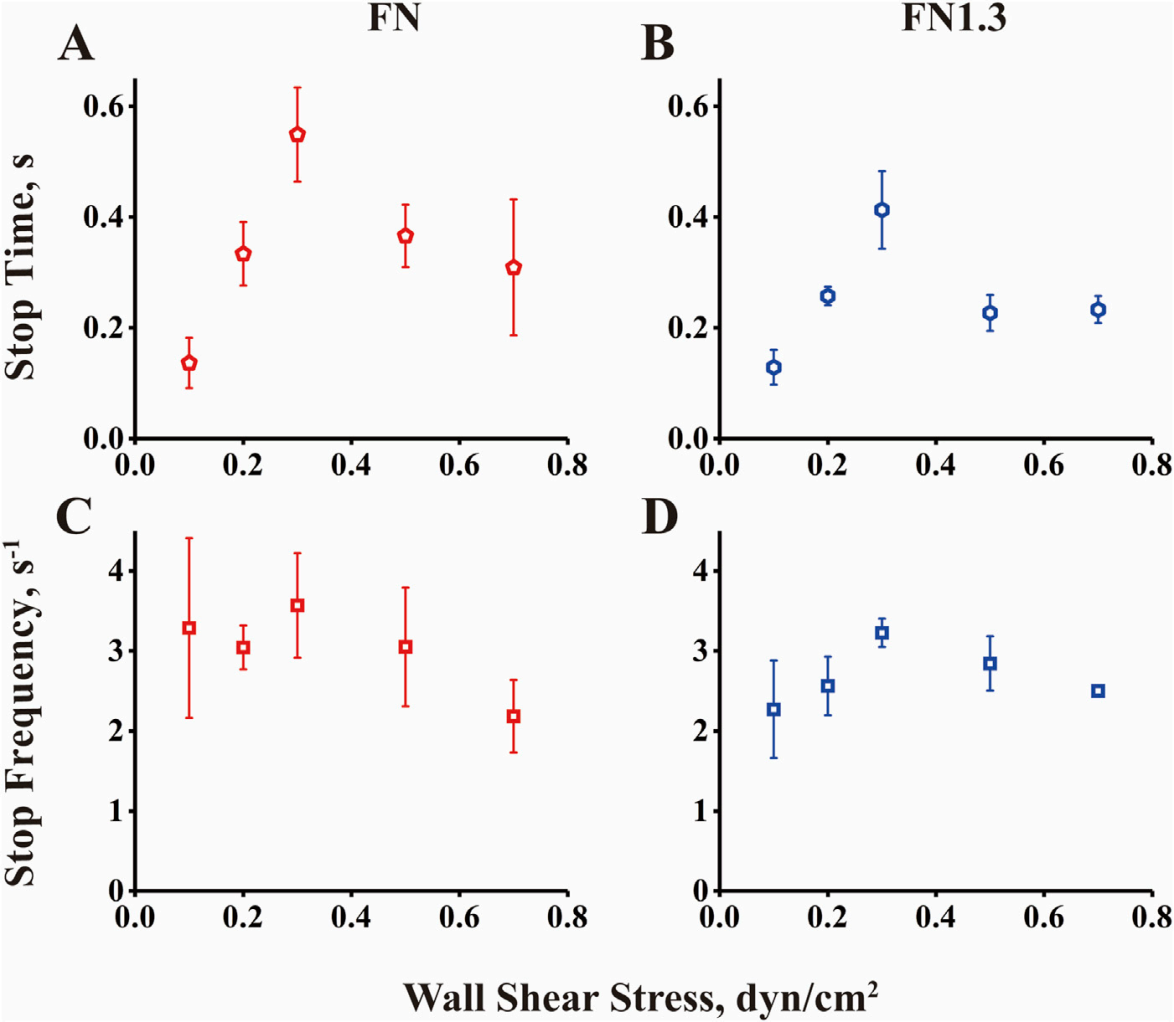
Rolling stop time and stop frequency of beads by interaction of integrin α_v_β_3_ with fibronectin (FN) or truncated fibronectin (FN1.3). **(A, B)** Mean stop times and **(C, D)** stop frequencies for FN/FN1.3-bearing microspheres rolling on substrate coated with integrin α_v_β_3_ (200 ng/mL) were plotted against wall shear stress. The data were recorded at 100 fps, and the mean ± SD of three independent experiments is presented.

To quantify the rolling behaviors shown in Figure 4, a homemade Excel macro was used to describe a minimum stop-and-go rolling model. A rolling step cycle includes a stop-and-go phase with or without a stop phase in the middle. We separated the whole rolling process into numerous stop and go phases from the instantaneous velocity curve when the acceleration and deceleration thresholds were set as 600 μm/s^2^. Thousands of events were gathered for approximately 15 microspheres during 10 s of rolling at each shear stress. Subsequently, we extracted the mean stop time and stop frequency as parameters to describe the rolling characteristics. As shown in Figure 5, the mean stop time and stop frequency first increased (from 0.1 to 0.3 dyn/cm^2^) and then decreased (from 0.3 to 0.7 dyn/cm^2^) when flow shear stress increased across the optimal value (0.3 dyn/cm^2^). This biphasic mean stop time derived from rolling of full/truncated FN-bearing beads on high density integrin α_v_β_3_-coated substrate is consistent with the biphasic transient tether lifetime measured at very low integrin α_v_β_3_ density. In addition, the curve of the reduced percentage of velocity (Figures 3C, D) matched the transient tether lifetime (Figures 2A, B) and mean stop time (Figures 5A, B), with similar biphasic curves and the same flow force optimum. This suggests that transitions between catch and slip bonds govern the lifetimes of transient tethers by single bonds and rolling tethers via a small number of bonds, and that they regulate rolling behaviors. In the catch bond regime, the increasing of force strengthens integrin α_v_β_3_-FN bonds to promote beads to stop, which increases the rolling regularity and reduces rolling velocity. Conversely, the force weakens integrin α_v_β_3_-FN bonds in the slip bond regime. In summary, the transitions between catch and slip bonds regulate rolling behavior.

### 3.6 The flow-enhanced microsphere adhesion to FN or FN1.3 mediated by integrin α_v_β_3_ is determined by wall shear stress rather than by fluid rate transport mechanisms

**FIGURE 6:**
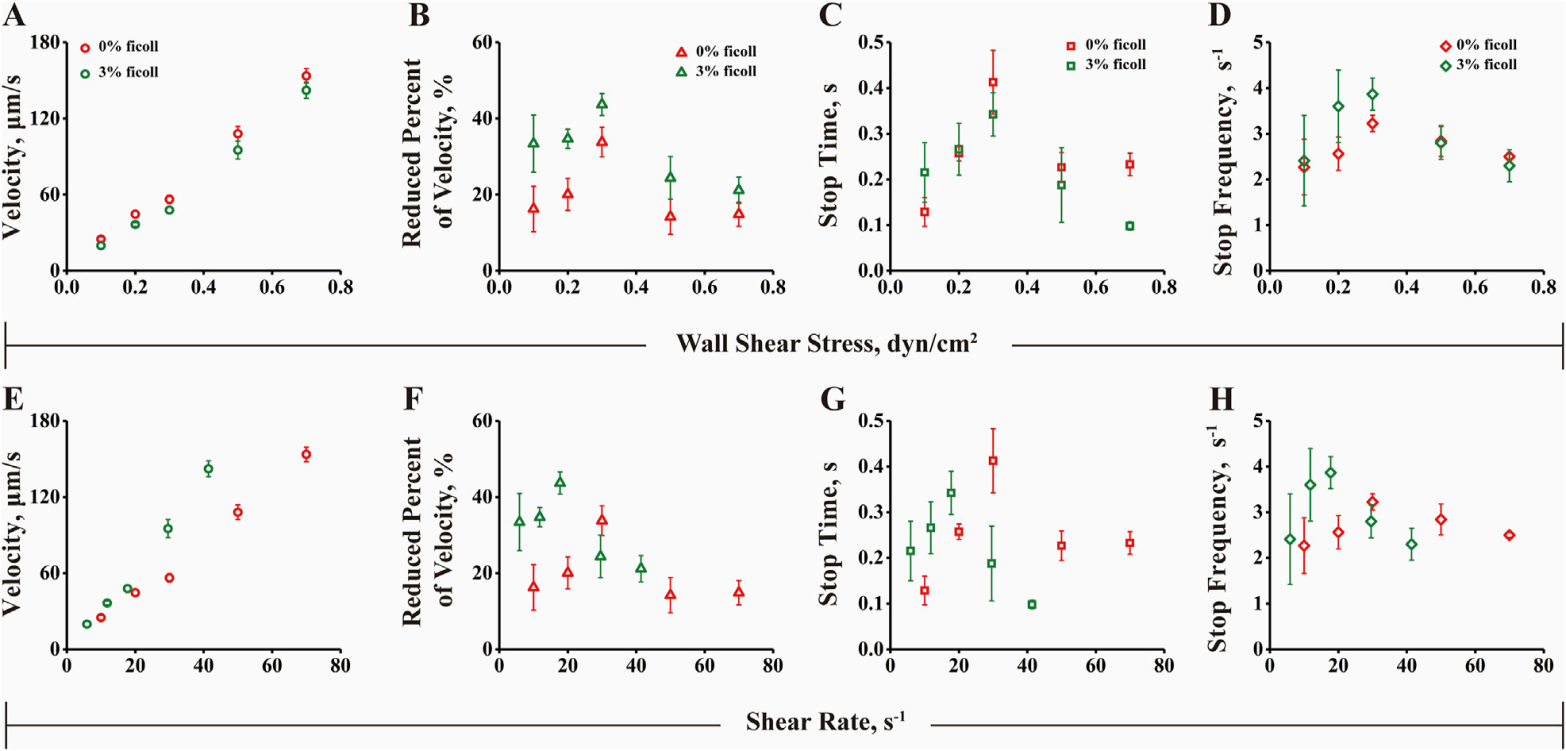
Rolling behavior analysis under wall shear stress and shear rate. **(A)** Rolling velocity, **(B)** Decrease rate, **(C)** Stop time and **(D)** Stop frequency of 0% or 3% ficoll as the ordinate and shear stress as the abscissa. **(E)** Rolling velocity, **(F)** Decrease rate, **(G)** Stop time and **(H)** Stop frequency of 0% or 3% ficoll as the ordinate and shear rate as the abscissa. The data were recorded at 100 fps, and the mean ± SD of three independent experiments is presented.

In a study on flow-enhanced adhesion mechanisms, it was suggested that the increase in molecular bond formation with increasing fluid shear force is due to the transport effect of the fluid, which increases the binding rate of the molecular bond (Yago et al., 2007). This results in an increased number of bonds being formed during cell or microsphere rolling. There are two possible explanations for this phenomenon. First, fluid transport can carry the ligands to the receptors, increasing the binding probability of the receptor and ligand and facilitating rapid bond formation and breakage. Second, the fluid shear force can flatten elastic cells and expand their contact area, leading to increased bond formation. To investigate whether the transport mechanism is involved in the adhesion of microspheres mediated by the α_v_β_3_-FN1.3 molecular bond, we added 3% (w/v) ficoll to the solution to change the viscosity of the fluid and repeated the rolling experiment under the same conditions. As shown in Figure 6, by comparing the rolling parameters of the microspheres in different adhesive fluids, it was found that the rolling parameters were not aligned under the shear rate coordinates, but a single curve was synthesized under the shear stress coordinates. This scaling relationship precludes the possibility that shear rate transport is a flow-enhanced rolling mechanism, suggesting that shear stress may be involved in the regulation of cell rolling behavior. These findings suggested that the flow-enhanced rolling mechanism is influenced by shear stress rather than the transport effect (Yago et al., 2007; Zhu et al., 2008).

## 4 Discussion

This study primarily utilized our laboratory’s PPFC system employing 6 μm microspheres as cell analogs to simulate rolling adhesion on vessels. The study investigated the force-regulated mechanisms of integrin α_v_β_3_-FN and integrin α_v_β_3_-FN1.3 interactions under varying shear forces. Initially, we verified if the microspheres’ adhesion was mediated by integrin α_v_β_3_-FN/FN1.3 interaction. Microspheres coated with FN or FN1.3 molecules were perfused within flow chambers pre-coated with PBS, 2% BSA, and 2% BSA + integrin α_v_β_3_. High-speed cameras were used to record the microsphere adhesion rates on these substrates at 100 fps. The results revealed greater physical adhesion on PBS and effective blockade of physical adhesion by 2% BSA, indicating microsphere adhesion on 2% BSA + α_v_β_3_-coated substrates were mediated by the integrin α_v_β_3_-FN/FN1.3 interaction. Further, we have performed flow chamber experiments using FN/FN1.3-coated microspheres at various concentrations to analyze the cell adhesion ratios of tethering and rolling. Our analysis indicated that the concentrations of 200 μg/mL FN and 100 μg/mL FN1.3 that we selected to coat microspheres were appropriate for investigating rolling behavior. At these concentrations, a portion of the cells can tether through single bonds, while others can roll via two bonds, providing an optimal scenario for analyzing rolling behavior for rolling cells using a minimal rolling model (Supplementary Figure S1). Subsequently, microspheres coated with FN/FN1.3 were perfused into flow chambers coated with α_v_β_3_ under different shear forces, and their adhesion behaviors were recorded. We employed IPP software to track the microsphere trajectories and obtained instantaneous velocity-time and accumulated distance-time graphs. Key parameters such as bond lifetime and dissociation rate of integrin α_v_β_3_-FN/FN1.3 were calculated. The results demonstrated that with increasing shear forces, the bond lifetime initially increased and then decreased, exhibiting force-dependent bimodal behavior (Figures 2A, E). Correspondingly, the dissociation rate of integrin α_v_β_3_-FN/FN1.3 exhibited a trend of first decreasing, then increasing with rising force (Figures 2B–D, F–H). This suggests that the interaction between integrin α_v_β_3_ and FN/FN1.3 has catch bonds at the level of single molecular bonds.

The study further investigated the rolling adhesion behavior of integrin α_v_β_3_-FN/FN1.3-mediated microspheres under different fluid shear forces. Differing from the previous section, rolling adhesion involved microsphere adhesion mediated by two or more integrin α_v_β_3_-FN/FN1.3 bonds (Yago et al., 2004; Zhu et al., 2008). To quantify the rolling behavior, we simplified it using a “stop-walk” model and analyzed the microsphere’s rolling behavior, deriving key parameters including average rolling velocity, average stop time, and average stop frequency (Yago et al., 2004). Results indicated that the rolling velocity of integrin α_v_β_3_-FN/FN1.3-mediated microspheres increased with escalating shear forces (Figures 2A, E). At first glance, there was no notable trend; however, when we changed the vertical coordinate from the rolling velocity to the percentage of reduced velocity, we found that as the wall shear stress increased, the percentage of velocity reduction first increased and then decreased, similar to the previous capture bond observations (Figures 3C, D). Moreover, the mean stop time initially increased and then decreased with shear force, in agreement with the trend observed for the bond lifetime (Figures 5A, B). Similarly, the average stop frequency first increased and then decreased with increasing shear force (Figures 5C, D).

To further explore whether the stop time and stop frequency of the microspheres increase first and then decrease with the increase of the fluid shear force and whether they are regulated by the “transport mechanism” of the fluid shear rate or by the force of the fluid shear stress, we added 3% (w/v) ficoll to the solution and assessed changes in fluid viscosity. The results were similar to previous reported findings (Yago et al., 2007), which ruled out shear rate transport as a mechanism for flow-enhanced rolling, but indicated that shear stress may be involved in regulating cell rolling (Figures 6A–H). In addition, we carried out concentration-dependent experiments to allow FN/FN1.3-coated microspheres to roll on substrates incubated with different concentrations of integrin α_v_β_3_ under the same shear stress of 0.3 dyn/cm^2^. The results showed that the rolling velocity of both FN- and FN1.3-coated microspheres progressively decreased (Supplementary Figures S2A, B), while the reduced percent in velocity increased (Supplementary Figures S2C, D) with the increase of integrin α_v_β_3_ concentration.

In previous studies on the molecular mechanism of L-selectin (Yago et al., 2004) and E-selectin (Li et al., 2016)-mediated enhancement of cell adhesion flow, the capture bond was found to increase rolling regularity by reducing rolling velocity. The similar studies on flow-enhanced cell adhesion are summarized in the Supplementary Table S1. In the current study, the form of a single-molecule catch bond was consistent with previous research results; however, in the multi-molecular form of the catch bond, the catch bond did not directly reduce the rolling velocity of the microsphere. However, with an increase in shear stress, the reduced percentage of velocity, stop time, and stop frequency showed force-related bimodal behavior. This indicates that our study also supports the theory of catch bond, and the rolling velocity does not decrease, which may be because the molecular density of integrin α_v_β_3_ on the bottom plate is insufficient to support continuous rolling of the microsphere. From the instantaneous velocity-time diagram (Figures 4B–F, H–L), it can be seen that the microsphere is not continuously rolling, but its rolling is composed of multiple instantaneous adhesion. In other words, the FN/FN1.3 on the microsphere does not recover immediately after the bond between FN/FN1.3 and the substrate integrin α_v_β_3_ molecule breaks, and FN/FN1.3 on the microsphere does not immediately combine with another α_v_β_3_ molecule on the bottom plate. Therefore, it does not involve continuous rolling. Because the microsphere does not continuously roll, and the stop time of the microsphere is a low proportion of the total time, the average rolling velocity of the microsphere is mainly determined by the transport rate. To reduce the influence of fluid transport, considering that the microsphere occupies a relatively small portion of the retention, we analyzed the velocity reduction ratio of the rolling microsphere relative to the free-floating microsphere under each shear force, which was calculated as follows: microsphere velocity reduction ratio = (free-floating microsphere velocity - rolling microsphere velocity)/free-floating microsphere velocity. This parameter represents the proportion of the residence time of the microspheres during the rolling process.

Integrin α_v_β_3_ is a focal molecule in tumor research, closely linked to tumor occurrence and progression. The α_v_ subunit within integrins has been confirmed to promote tumor angiogenesis, a pivotal event in tumor development. These integrins promote the survival of tumor cells under fluid shear forces by interacting with the extracellular matrix. Specifically, integrin α_v_β_3_ primarily interacts with FN molecules in the extracellular matrix, fostering tumor cell survival and invasion. Therefore, understanding the interactions between integrin α_v_β_3_ and their ligands is crucial for comprehending tumor occurrence and development and designing targeted therapies for these integrins in cancer treatment. Besides, in the study of Elosegui-Artola et al., atomic force microscopy and micropipette technology were used to measure the lifetime of a single fibronectin bond with α_v_β_3_, and it was found that there was a catch bond between fibronectin and α_v_β_3_ (Elosegui-Artola et al., 2016). In our study, we used PPFC to explore integrin α_v_β_3_ receptor, FN1.3, and further investigated the rolling event, which is a good complement to the study of integrin α_v_β_3_-FN interaction. In addition, the experimental setup using FN/FN1.3-coated microspheres interacting with integrin α_v_β_3_-functionalized plates does not fully mimic the *in vivo* environment; it only provides a platform to investigate α_v_β_3_-FN/FN1.3 interactions. Future studies should aim to employ α_v_β_3_-coated microspheres on FN/FN1.3-coated substrates to more accurately approximate physiological interactions and further substantiate our findings.

This study demonstrates that the interaction between integrin α_v_β_3_ and fibronectin is a dynamic process highly sensitive to fluid shear stress. By utilizing a PPFC, we have uncovered a biphasic pattern in the adhesive behavior of fibronectin-functionalized microspheres on integrin α_v_β_3_ coated substrates. This behavior, characterized by an initial increase followed by a decrease in bond lifetimes with increasing shear forces, signifies a transition from “catch bond” to “slip bond” phenomenon. This transition is crucial for tumor cells to modulate their adhesion in low shear stress regions, facilitating migration. Our findings enhance the understanding of tumor cell mechanics and suggest a potential therapeutic approach by targeting the “catch bond” property of integrin α_v_β_3_ under specific shear conditions. This could inhibit tumor cell adhesion and migration, presenting a novel strategy in cancer treatment.

## Data Availability

The datasets presented in this study can be found in online repositories. The names of the repository/repositories and accession number(s) can be found in the article/Supplementary Material.
