# Supplementary material for: Interaction of integrin αvβ3 and fibronectin under fluid shear forces: implications for tumor cell adhesion and migration
Source: Front Cell Dev Biol. 2025 Feb 13;13:1512672. doi: 10.3389/fcell.2025.1512672 (PMC11894259; doi:10.3389/fcell.2025.1512672)
Supplement: Supplementary file 1 [file DataSheet1.pdf]

Supplementary Material

1 Supplementary Figures and Table

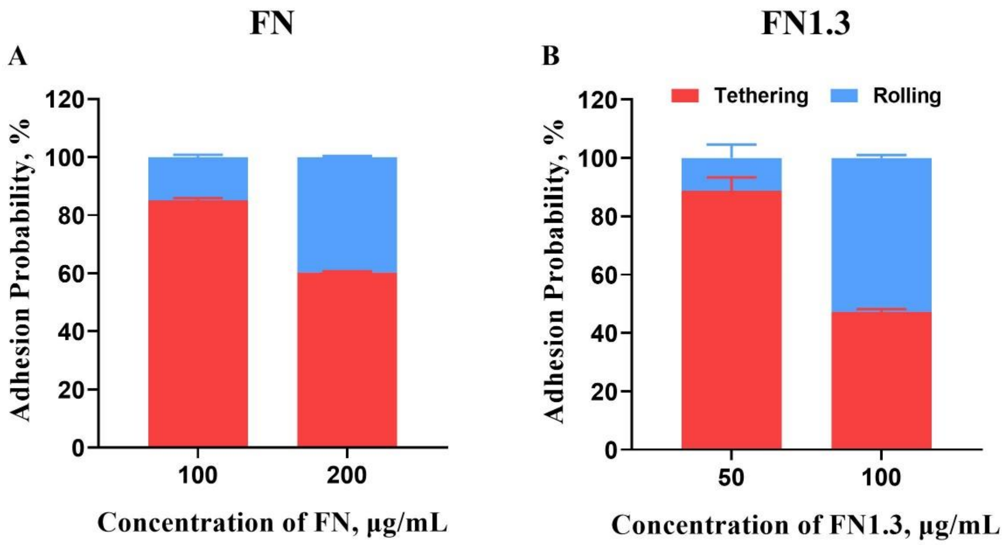

Figure S1. The adhesion probability of microspheres. The adhesion probability of microspheres coated with different concentrations of FN (A) and FN1.3 (B) on integrin $\alpha_v\beta_3$-functionalized plates.

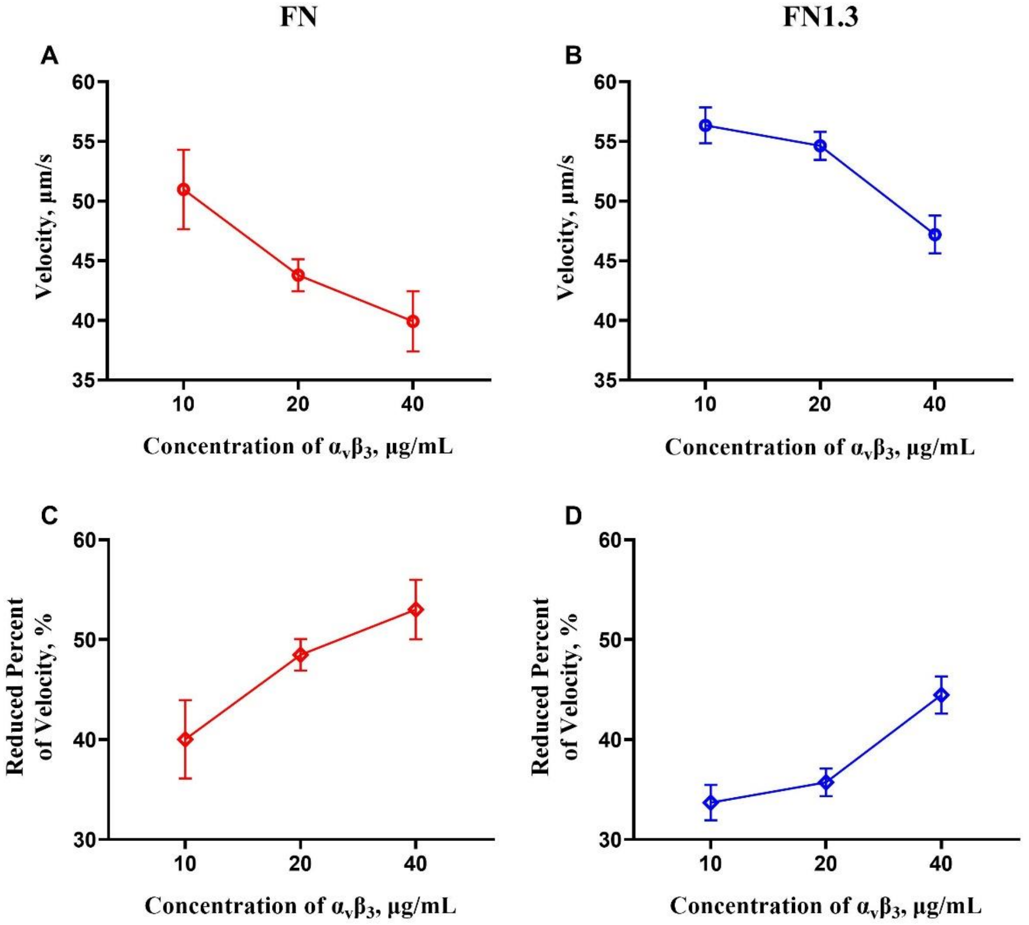

Figure S2. The influence of $\alpha_v\beta_3$ concentration on rolling velocity. (A-B) Rolling velocities of FN and FN1.3-coated microspheres on substrates incubated with different $\alpha_v\beta_3$ concentrations. (C-D) Reduced percent of velocity of FN and FN1.3-coated microspheres on substrates incubated with varying $\alpha_v\beta_3$ concentrations.

Table S1. Summary of force-enhanced adhesion studies

Year	Receptor	Ligand	Function	Shear force	Threshold	Journal	References
1996	L-selectin	PNAd	Leukocyte adhesion	0.1-10 dyn/cm^2	0.8 dyn/cm^2	*Nature*	(Finger, et al.,1996)
1996	GPIb	VWF	Platelet adhesion	50-1500 s^{-1}	600-900 s^{-1}	*Cell*	(Savage, et al.,1996)
2002	FimH	Mannose	Bacterium adhesion	0.01-10 dyn/cm^2	0.5 dyn/cm^2	*Cell*	(Thomas, et al.,2002)
2003	P-selectin	PSGL-1	Leukocyte adhesion	0-0.6 dyn/cm^2	0.2-0.3	*Nature*	(Marshall, et al.,2003)
2005	P-selectin	PSGL-1	Leukocyte adhesion	0-30 pN	10 pN	*PNAS*	(Barsegov, et al.,2005)
2009	$\alpha_5\beta_1$	Fibronectin	Cell anchor to ECM	0-60 pN	20-30 pN	*J Cell Biol*	(Kong, et al.,2009)
2010	Streptavidin	Biotin	/	12-24 pN	14 pN	*Nanotechnology*	(Jeney, et al.,2010)
2010	Kinetochore	Microtubule	Cell division	0-16 pN	3 pN	*Nature*	(Akiyoshi, et al.,2010)
2010	$\alpha_L\beta_2$	ICAM-1	Leukocyte adhesion	0-30 pN	10 pN	*J Biol Chem*	(Chen, et al.,2010)
2010	GPIb	VWF	Platelet adhesion	0-40 pN	9.55-10.58 pN	*Nature*	(Kim, et al.,2010)
2012	CD44	Hyaluronans	/	0.2-1 dyn/cm^2	0.55 dyn/cm^2	*Histochem Cell*	(Richter, et al.,2012)
2014	TCR	pMHC	T cell recognition of antigen	0-30 pN	10 pN	*Trends Immunol*	(Depoil and
2017	Fibronectin	/	Stabilizes Borrelia	0.5-3 dyn/cm^2	1 dyn/cm^2	*PNAS*	(Niddam, et al.,2017)
2022	TCR	pMHC	T cell recognition of antigen	0-50 pN	10 pN	*Science*	(Zhao, et al.,2022)
2022	TCR&CD4	pMHC	Antigen-presenting	0-35 pN	15 pN	*Nature*	(Rushdi, et al.,2022)
2022	α-β-catenin	F-actin	Actin cytoskeleton adhesion	0-80 pN	5 pN	*Nature*	(Arbore, et al.,2022)
2023	$\alpha_4\beta_7$	MAdCAM-1	Leukocyte adhesion	0.1-0.6 dyn/cm^2	0.3-0.4	*Int J Mol Sci*	(Su, et al.,2023)
2023	CD44	Hyaluronans	/	0-35 pN	20 pN	*Int J Mol Sci*	(Yao, et al.,2023)
2024	CD40	CD40L	Prevent B cell silencing	0-40 pN	15 pN	*Science Advances*	(Choi, et al.,2024)
2024	PD-1	PD-L1/2	Immune checkpoint receptor	0-25 pN	8 pN	*Nature*	(Li, et al.,2024)

References:

AKIYOSHI, B., SARANGAPANI, K. K., POWERS, A. F., NELSON, C. R., REICHOW, S. L., ARELLANO-SANTOYO, H., GONEN, T., RANISH, J. A., ASBURY, C. L. & BIGGINS, S. 2010. Tension directly stabilizes reconstituted kinetochore-microtubule attachments. *Nature,* 468**,** 576-U255.

ARBORE, C., SERGIDES, M., GARDINI, L., BIANCHI, G., KASHCHUK, A. V., PERTICI, I., BIANCO, P., PAVONE, F. S. & CAPITANIO, M. 2022. α-catenin switches between a slip and an asymmetric catch bond with F-actin to cooperatively regulate cell junction fluidity. *Nature Communications,* 13(1)**,** 1146.

BARSEGOV, V. & THIRUMALAI, D. 2005. Dynamics of unbinding of cell adhesion molecules: Transition from catch to slip bonds. *Proceedings of the National Academy of Sciences of the United States of America,* 102**,** 1835-1839.

CHEN, W., LOU, J. Z. & ZHU, C. 2010. Forcing Switch from Short- to Intermediate- and Long-lived States of the αA Domain Generates LFA-1/ICAM-1 Catch Bonds. *Journal of Biological Chemistry,* 285**,** 35967-35978.

CHOI, H. K., TRAVAGLINO, S., MüNCHHALFEN, M., GöRG, R., ZHONG, Z., LYU, J., REYES-AGUILAR, D. M., WIENANDS, J., SINGH, A. & ZHU, C. 2024. Mechanotransduction governs CD40 function and underlies X-linked hyper-IgM syndrome. *Science Advances,* 10(46)**,** eadl5815.

DEPOIL, D. & DUSTIN, M. L. 2014. Force and affinity in ligand discrimintion by the TCR. *Trends in Immunology,* 35**,** 597-603.

FINGER, E., KD., P., R., A., MB., L., UH., V. A. & TA., S. 1996. Adhesion through L-selectin requires a threshold hydrodynamic shear. *Nature,* 379(6562)**,** 266-269.

JENEY, S., MOR, F., KOSZALI, R., FORRó, L. & MOY, V. T. 2010. Monitoring ligand-receptor interactions by photonic force microscopy. *Nanotechnology,* 21(5)**,** 255102.

KIM, J., ZHANG, C. Z., ZHANG, X. H. & SPRINGER, T. A. 2010. A mechanically stabilized receptor-ligand flex-bond important in the vasculature. *Nature,* 466**,** 992-U123.

KONG, F., GARCíA, A. J., MOULD, A. P., HUMPHRIES, M. J. & ZHU, C. 2009. Demonstration of catch bonds between an integrin and its ligand. *Journal of Cell Biology,* 185**,** 1275-1284.

LI, K., CARDENAS-LIZANA, P., LYU, J., KELLNER, A. V., LI, M., CONG, P., WATSON, V. E., YUAN, Z., AHN, E., DOUDY, L., LI, Z., SALAITA, K., AHMED, R. & ZHU, C. 2024. Mechanical force regulates ligand binding and function of PD-1. *Nature Communications,* 15(1)**,** 8339.

MARSHALL, B. T., LONG, M., PIPER, J. W., YAGO, T., MCEVER, R. P. & ZHU, C. 2003. Direct observation of catch bonds involving cell-adhesion molecules. *Nature,* 423**,** 190-193.

NIDDAM, A. F., EBADY, R., BANSAL, A., KOEHLER, A., HINZ, B. & MORIARTY, T. J. 2017. Plasma fibronectin stabilizes-endothelial interactions under vascular shear stress by a catch-bond mechanism. *Proceedings of the National Academy of Sciences of the United States of America,* 114**,** E3490-E3498.

RICHTER, U., WICKLEIN, D., GELEFF, S. & SCHUMACHER, U. 2012. The interaction between CD44 on tumour cells and hyaluronan under physiologic flow conditions: implications for metastasis formation. *Histochemistry and Cell Biology,* 137**,** 687-695.

RUSHDI, M. N., PAN, V., LI, K. T., CHOI, H. K., TRAVAGLINO, S., HONG, J. S., GRIFFITTS, F., AGNIHOTRI, P., MARIUZZA, R. A., KE, Y. G. & ZHU, C. 2022. Cooperative binding of T cell receptor and CD4 to peptide-MHC enhances antigen sensitivity. *Nature Communications,* 13(1)**,** 7055.

SAVAGE, B., E., S. & ZM., R. 1996. Initiation of platelet adhesion by arrest onto fibrinogen or translocation on von Willebrand factor. *Cell,* 84(2)**,** 289-297.

SU, Y. M., LUO, Z. Q., SUN, D. S., YANG, B. S. & LI, Q. H. 2023. The Force-Dependent Mechanism of an Integrin α4β7-MAdCAM-1 Interaction. *International Journal of Molecular Sciences,* 24(22)**,** 16062.

THOMAS, W. E., TRINTCHINA, E., FORERO, M., VOGEL, V. & SOKURENKO, E. V. 2002. Bacterial adhesion to target cells enhanced by shear force. *Cell,* 109**,** 913-923.

YAO, Z. Y., WU, J. H. & FANG, Y. 2023. Moderate Constraint Facilitates Association and Force-Dependent Dissociation of HA-CD44 Complex. *International Journal of Molecular Sciences,* 24(3)**,** 2243.

ZHAO, X., KOLAWOLE, E. M., CHAN, W. P., FENG, Y. N., YANG, X. B., GEE, M. H., JUDE, K. M., SIBENER, L. V., FORDYCE, P. M., GERMAIN, R. N., EVAVOLD, B. D. & GARCIA, K. C. 2022. Tuning T cell receptor sensitivity through catch bond engineering. *Science,* 376(6589)**,** eabl5282.
